# Deletion of *PKBα*/*Akt1* Affects Thymic Development

**DOI:** 10.1371/journal.pone.0000992

**Published:** 2007-10-03

**Authors:** Elisabeth Fayard, Jason Gill, Magdalena Paolino, Debby Hynx, Georg A. Holländer, Brian A. Hemmings

**Affiliations:** 1 Friedrich Miescher Institute for Biomedical Research, Basel, Switzerland; 2 Pediatric Immunology, Center for Biomedicine, Department of Clinical-Biological Sciences, The University of Basel, The University Children's Hospital, Basel, Switzerland; Oklahoma Medical Research Foundation, United States of America

## Abstract

**Background:**

The thymus constitutes the primary lymphoid organ for the majority of T cells. The phosphatidyl-inositol 3 kinase (PI3K) signaling pathway is involved in lymphoid development. Defects in single components of this pathway prevent thymocytes from progressing beyond early T cell developmental stages. Protein kinase B (PKB) is the main effector of the PI3K pathway.

**Methodology/Principal Findings:**

To determine whether PKB mediates PI3K signaling in the thymus, we characterized *PKB* knockout thymi. Our results reveal a significant thymic hypocellularity in *PKBα* ^−/−^ neonates and an accumulation of early thymocyte subsets in *PKBα* ^−/−^ adult mice. Using thymic grafting and fetal liver cell transfer experiments, the latter finding was specifically attributed to the lack of PKBα within the lymphoid component of the thymus. Microarray analyses show that the absence of PKBα in early thymocyte subsets modifies the expression of genes known to be involved in pre-TCR signaling, in T cell activation, and in the transduction of interferon-mediated signals.

**Conclusions/Significance:**

This report highlights the specific requirements of PKBα for thymic development and opens up new prospects as to the mechanism downstream of PKBα in early thymocytes.

## Introduction

The thymus constitutes the primary lymphoid organ for the majority of T cells as its microenvironments provide an exclusive combination of different stromal cell types critical for the generation and selection of thymocytes to mature T cells [1]. During their thymic development, T lineage committed precursors progress through an ordered sequence of differentiation events [2]. These events reflect the complex progression from immature progenitors to post-selection T cells, which are tolerant to self but recognize foreign antigens in the context of self-MHC molecules. Immature intrathymic precursors are characterized by the absence of CD4 and CD8 cell surface expression and are hence designated double negative (DN) thymocytes. Based on the expression of CD25 and CD44, DN thymocytes are further distinguished into four sequentially evolving subpopulations (DN1-DN4) [3]. Early during maturation, the productive rearrangement of the T cell antigen receptor β (*TCRβ*) locus allows for the expression of a nascent TCRβ chain that, together with the expression of the pre-Tα (pTα) chain and the CD3 complex, forms the pre-TCR complex [4]. This particular stage represents a critical checkpoint in T cell development that is known as β-selection. Signaling via a functional pre-TCR allows for the further differentiation of thymocytes and initiates the surface expression of both CD4 and CD8. Developing T cells concurrently expressing CD4 and CD8 (designated double positive, DP, thymocytes) rearrange their *TCRα* locus, which enables the cell surface expression of a mature TCRαβ complex. Subsequently, the events of positive and negative TCR selection take place giving rise to single CD4- or CD8-positive (SP) mature T cells that are eventually released into the periphery [5]. Changes in the thymic stromal compartment and alterations of key signaling pathways in thymocytes result in an aberrant development and the lack of regular T cells.

The phosphatidyl-inositol 3 kinase (PI3K) signaling pathway has been reported to be involved in lymphoid development as impaired PI3K signaling results in immunodeficiency, while unrestrained signaling contributes to lymphoma formation and autoimmunity [6]. The function of PI3K is to convert at the plasma membrane phosphatidyl-inositol-(4,5)-bisphosphate (PIP2) to the second messenger phosphatidyl-inositol-(3,4,5)-trisphosphate (PIP3). The 3′-phosphate lipid phosphatase PTEN antagonizes the generation of PIP3 [7]. PIP3 acts as a binding site for various intracellular enzymes that contain a pleckstrin homology (PH) domain, such as the serine/threonine kinases phosphoinositide-dependent kinase 1 (PDK1) and protein kinase B (PKB). Hence, PIP3 promotes the translocation of the corresponding proteins from the cytoplasm to the plasma membrane. Recruited at the membrane, PDK1 phosphorylates a key residue within the catalytic domain of one of its substrates, PKB [8], which is the most important effector of the PI3K pathway. To be fully active, PKB needs to be phosphorylated at a second key residue located in the hydrophobic motif within the regulatory domain [9]. For this to occur, a number of upstream kinase candidates have been identified, including DNA-dependent protein kinase (DNA-PK) [10] or the rictor-mTOR complex [11]. Once activated, PKB phosphorylates numerous substrates influencing diverse cellular and physiological processes attributed to the PI3K pathway [12].

Mice genetically impaired for single components of the PI3K signaling pathway display distinct deficiencies in the development and function of the immune system. For instance, severe combined immunodeficiency (SCID) in mice correlates with a nonsense mutation within the gene of the DNA-PK catalytic subunit (*DNA-PKcs*) [13]–[15]. Moreover, mice deficient for *DNA-PKcs* exhibit a severe immunodeficiency partly associated with a block in T cell development due to impaired variable/diversity/joining (VDJ) rearrangements at the DN3 stage [16]. Furthermore, deletion of *PDK1* in T cell precursors prevents T cell differentiation at the DN to DP transition and downregulates the cell size of immature thymocytes [17], suggesting that signals downstream of PDK1 and/or DNA-PK are essential for T cell development. On the other hand, heterozygous deletion of *PTEN* and T cell-specific *PTEN*-null mutation in mice lead to increased thymic cellularity and the development of not only lymphoid hyperplasia, which progresses to T cell lymphoma, but also autoimmunity likely due to impaired Fas signaling [18]–[21]. Mutations in *PTEN* allow unrestrained PIP3 production, which results in constitutive PKB activation. Correspondingly, mice engineered to express a constitutively active form of PKB in T cells display a phenotype similar to that of *PTEN*-mutant mice [22]–[24].

Three PKB isoforms encoded by separate genes and of identical structural organization have been described for mammalian cells: PKBα, PKBβ, and PKBγ [25]. While PKBα is ubiquitously detected, PKBβ and PKBγ tend to be expressed in a tissue-specific pattern. Targeted disruption of each of these isoforms in mice has helped to elucidate the physiological *in vivo* relevance of the PKB isoforms, revealing both specific and redundant functions [26]–[34]. However, specific immunological defects have not been reported for single mutant mice.

To characterize the specific contribution of distinct PKB isoforms within the PI3K signaling pathway for thymic development, we investigated mice deficient for each of the isoforms of PKB. Our results reveal a significant thymic hypocellularity in *PKBα* ^−/−^ neonates and an accumulation of early thymocyte subsets at the DN to DP transition during adult T cell development in *PKBα* ^−/−^ mice due to cell-autonomous effects. Moreover, in early thymocytes PKBα regulates genes known to respond to pre-TCR, TCR, or interferon signaling. This report uncovers the specific requirements of PKBα for thymic development.

## Results

### The deletion of *PKBα* leads to a hypocellular thymus in mouse neonates

**Figure 1:**
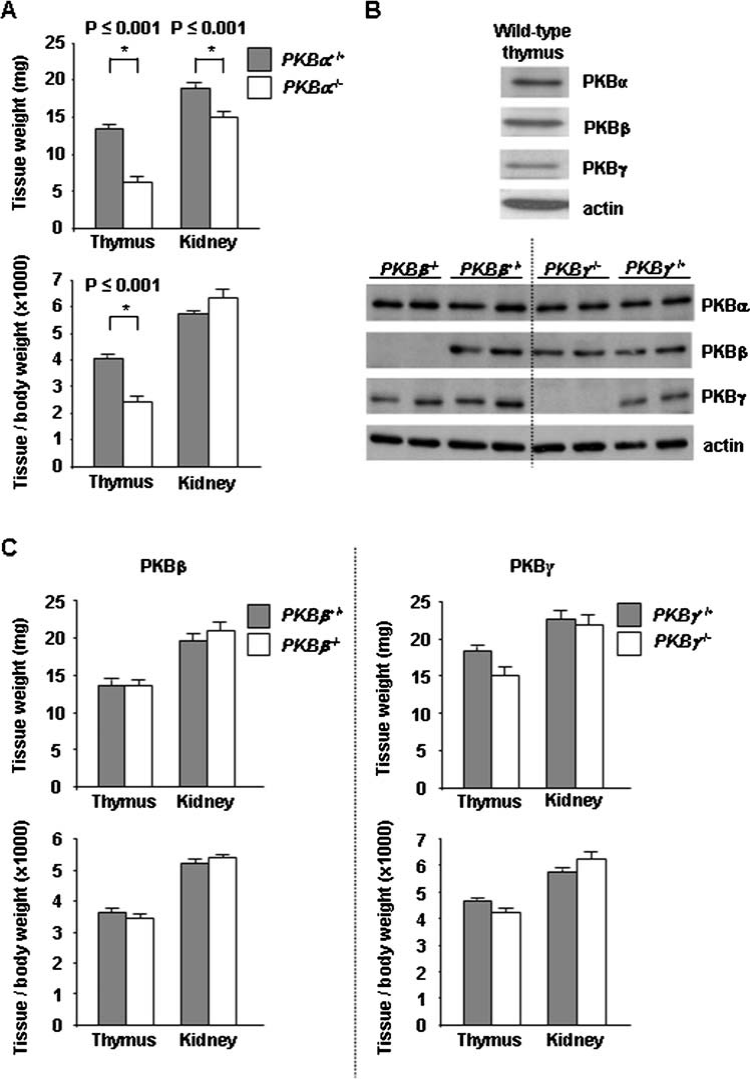
The deletion of *PKBα* leads to a reduced thymic size in mouse neonates. A: The weight of freshly dissected thymi was measured in *PKBα* ^+/+^ and *PKBα* ^−/−^ neonates (top panel) and expressed as ratio to body weight (bottom panel). The kidney was used as a control. Error bars represent standard error of the mean; n≥13. B: Western-blot analysis of 50 µg protein extracts from wild-type neonatal thymus using PKB isoform specific antibodies (top panel). Western-blot analysis of 50 µg protein extracts from *PKBβ* ^−/−^, *PKBβ* ^+/+^, *PKBγ* ^−/−^, and *PKBγ* ^+/+^ neonatal thymi using PKB isoform specific antibodies (bottom panel). Actin was used as a loading control. C: The weight of freshly dissected thymi was measured in *PKBβ* ^+/+^, *PKBβ* ^−/−^, *PKBγ* ^+/+^, and *PKBγ* ^−/−^ neonates (top panels) and expressed as ratio to body weight (bottom panels). The kidney was used as a control. Error bars represent standard error of the mean. n≥7 (n = number of mice analyzed per genotype).

To determine the potential impact of PKB on thymic development, we analyzed the thymus of *PKB* mutant mice. The dissection of neonates revealed that the size of *PKBα* ^−/−^ thymi was reduced to less than half that of wild-type controls (Figure 1A, top panel). We and others had previously reported that genetic ablation of *PKBα* leads to a decreased body weight [26], [28], [34], suggesting a general but proportional reduction in the size of any organ. To confirm this, we compared the weight of the thymus in relation to the body weight. In neonatal mice deficient for *PKBα*, the thymus weight was reduced to 60% of wild-type controls when normalized to the body weight (Figure 1A, bottom panel). This finding was specific since the weight of other organs, such as the kidney, was reduced in proportion to the reduction of body weight (Figure 1A and data not shown). In contrast to the results in neonatal mice, the relative weight of the thymus was not diminished in adult animals deficient for *PKBα* (Figure S1A), a result that is consistent with our previous findings [34].

Western-blot analyses showed that all three PKB isoforms were present within the thymus of wild-type neonates (Figure 1B, top panel), rendering it possible that a deletion of either *PKBβ* or *PKBγ* could also affect thymic size. Mice deficient for either of these isoforms demonstrated, however, a normal thymus weight (Figure 1C). Moreover, the loss of one of the PKB isoforms was not compensated by an upregulation in the expression of any of the other isoforms (Figure 1B, bottom panel). Taken together, these data indicate that the loss of expression of a PKB isoform is not off set by higher expression levels of another isoform and that PKBα is necessary for the normal size of the neonatal thymus.

**Figure 2:**
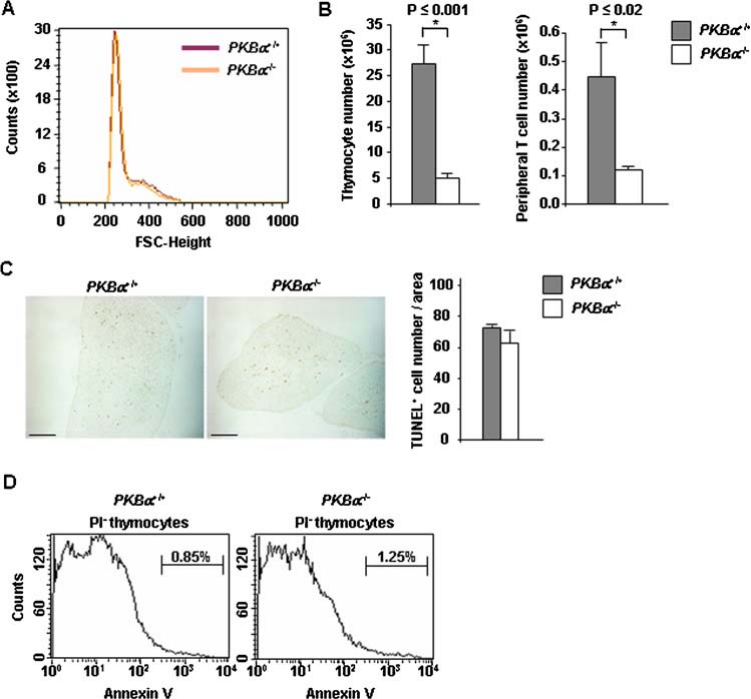
The deletion of *PKBα* leads to a reduced number of thymocytes in neonatal mice. A: Thymocytes were isolated from neonatal *PKBα* ^+/+^ and *PKBα* ^−/−^ littermates and their size compared by flow cytometry using the forward scatter (FSC) parameter. The histogram is representative of 3 litters. B: (left panel) Thymocytes were isolated and counted from *PKBα* ^+/+^ and *PKBα* ^−/−^ neonatal mice. (right panel) Lymphocytes isolated from the spleen of *PKBα* ^+/+^ and *PKBα* ^−/−^ neonates were stained with anti-CD19 and anti-CD3 antibodies. The number of T cells (CD3^+^CD19^−^) is shown. n≥3. Error bars represent standard error of the mean. C: TUNEL assay on neonatal thymus sections from *PKBα* ^+/+^ and *PKBα* ^−/−^ littermates. The graph represents the quantification of TUNEL-positive cells from 5 fields on 3 sections. The result shown is representative of 3 independent experiments. The bar shown on the pictures represents 200 µm. Error bars represent standard error of the mean. D: Thymocytes were isolated from *PKBα* ^+/+^ and *PKBα* ^−/−^ neonates and stained with annexin V and propidium iodide (PI). Histograms show results that are representative of 2 independent experiments; n≥3 (n = number of mice per genotype within the same experiment).

The organ size is determined by the number and/or the volume of its cells. While the size of thymocytes was not affected by the loss of *PKBα* (Figure 2A), the number of *PKBα* ^−/−^ thymocytes was significantly reduced in newborns (but not in adults) when compared to that of wild-type littermates (Figure 2B, left panel, and S1B). Hence, a lower thymocyte cellularity accounted, in neonatal mice, for the diminished tissue weight and also correlated with a decrease in peripheral T cells (Figure 2B, right panel). To determine whether the decreased thymic cellularity of neonatal mice was caused by an increase in programmed cell death, we performed TUNEL assay on thymus tissue sections as well as annexin V/propidium iodide staining of thymocytes. The frequency of apoptotic cells within the thymus was similar for control and *PKBα* ^−/−^ neonates, excluding the possibility of increased programmed cell death to account for the noted hypocellularity (Figure 2C and 2D).

### The lack of PKBα leads to an accumulation of thymocyte subsets at an early checkpoint during T cell development

**Figure 3:**
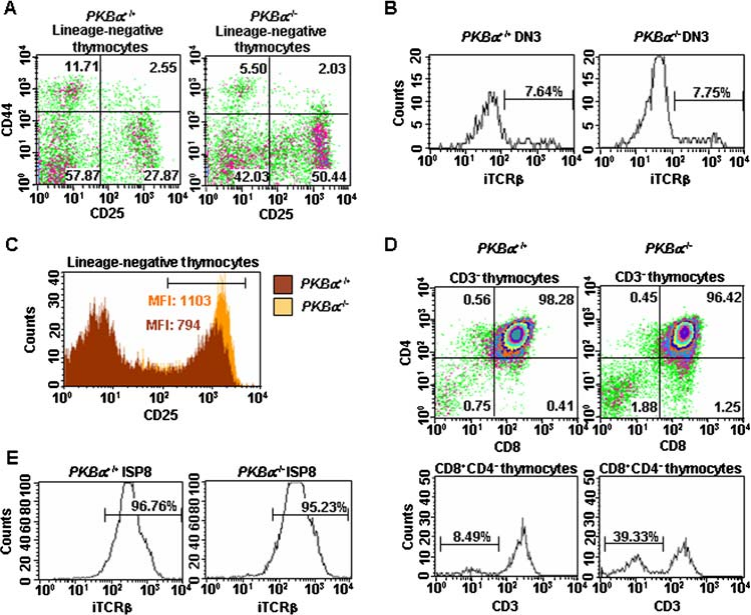
The lack of PKBα leads to an accumulation of DN3 and ISP8 early thymocyte subsets. Flow cytometric analysis of early thymocytes at the transition from DN to DP. A: Density plots show thymocytes from *PKBα* ^+/+^ and *PKBα* ^−/−^ mice that were stained with cell surface markers for identification of lineage-negative thymocytes DN1 (CD25^−^CD44^+^), DN2 (CD25^+^CD44^+^), DN3 (CD25^+^CD44^−^), and DN4 (CD25^−^CD44^−^). B: Histograms show the intracellular protein expression of TCRβ (iTCRβ) in DN3 thymocytes from *PKBα* ^+/+^ and *PKBα* ^−/−^ mice. C: Histograms show the surface expression of CD25 on lineage-negative *PKBα* ^+/+^ and *PKBα* ^−/−^ thymocytes. MFI: mean fluorescence intensity. D: Density plots and histograms show thymocytes from *PKBα* ^+/+^ and *PKBα* ^−/−^ mice that were labeled with cell surface markers for identification of ISP8 (CD4^−^CD8^+^CD3^−^) thymocytes. E: Histograms show the intracellular protein expression of TCRβ (iTCRβ) in ISP8 thymocytes from *PKBα* ^+/+^ and *PKBα* ^−/−^ mice. The results shown are representative of 3 independent experiments on 4 to 6 week-old mice. n≥4 (n = number of mice per genotype within the same experiment).

To address whether a partial or complete block in T cell development could explain the hypocellularity observed in the thymus of neonates deficient for *PKBα*, we analyzed in *PKBα* ^+/+^ and *PKBα* ^−/−^ mice the major thymocyte subsets. Using flow cytometry, the main subsets of mutant mice displayed similar relative frequencies when compared to age-matched wild-type controls in both neonatal and adult mice (data not shown and Figure S2A). We therefore excluded that a block in T cell development would account for thymic hypocellularity in *PKBα* ^−/−^ neonates. However, a refined phenotypic analysis of adult thymocytes revealed an accumulation at early developmental stages, suggesting that, in addition to its effect on neonatal thymic cellularity, the deletion of *PKBα* also affected T cell development. Even though CD25^−^CD44^+^ cell subset (designated DN1) appeared to be reduced in *PKBα* ^−/−^ mice (Figure 3A), when analyzed for surface expression of c-kit, T cell precursors (CD25^−^CD44^+^c-kit^+^) were only slightly affected (data not shown). On the other hand, while CD25^+^CD44^+^ (designated DN2) cell subset was unchanged, a subpopulation of thymocytes that express CD25 but lack CD44 at the cell surface (defined as DN3) was increased in the adult *PKBα* ^−/−^ thymus in comparison to wild-type controls (Figure 3A). These DN3 thymocytes are at a developmental stage immediately prior to the β-selection checkpoint. DN3 thymocytes with a productively rearranged *TCRβ* locus and a successful expression of the pre-TCR complex pass the β selection checkpoint, downregulate CD25, and develop into thymocytes with a DN4 phenotype (CD25^−^CD44^−^). In view of an accumulation of DN3 cells in *PKBα* ^−/−^ mice, we investigated whether it could be associated with a defect in *TCRβ* expression. We measured intracellular TCRβ protein using flow cytometry and found the expression of this receptor subunit in DN3 thymocytes at comparable levels in both *PKBα* ^−/−^ and control mice (Figure 3B). Furthermore, *PKBα* ^+/+^ and *PKBα* ^−/−^ DN4 thymocytes expressed intracellularly the TCRβ proteins (Figure S2B). These results suggest that the *PKBα* deletion does not impair the rearrangement or the expression of the *TCRβ* chain and that PKBα is not directly involved in the process of pre-TCR formation. However, the cell surface expression of the α chain of the interleukin-2 receptor (CD25) was increased among DN3 cells of *PKBα* ^−/−^ mice when compared to the equivalent subpopulation of wild-type mice, suggesting a role of PKBα in cell signaling at this stage of early thymocyte development (Figure 3C). Moreover, a population of immature thymocytes expressing CD8, but still lacking the cell surface expression of both CD4 and CD3, and displaying intracellular TCRβ proteins, accumulated in the thymus of *PKBα* ^−/−^ mutant mice (Figure 3D and 3E). These thymocytes represent a stage immediately prior to that of DP cells and are hence designated immature single CD8^+^ thymocytes (ISP8) [35]. However, no apparent differences in thymocyte proliferation, apoptosis, or size were detected when comparing *PKBα* ^+/+^ and *PKBα* ^−/−^ specific thymocyte subsets (Figure S2C and S2D and data not shown). Overall, our data reveal a critical role for PKBα in the transition from a DN to DP phenotype with a partial accumulation of DN3 and ISP8 thymocytes in mice deficient for *PKBα* expression.

### The accumulation of thymocyte subsets at the DN to DP transition in early T cell development originates from the absence of PKBα in hematopoietic precursors

**Figure 4:**
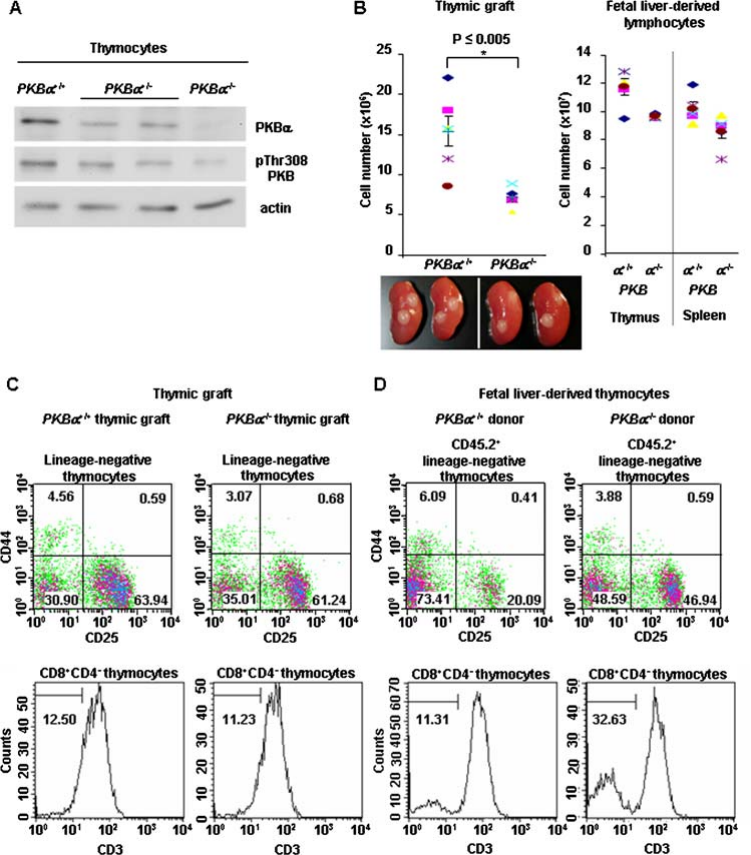
The accumulation of early thymocytes is due to PKBα deficiency in the lymphoid compartment. A: Western-blot analysis of 50 µg protein extracts from *PKBα* ^+/+^, *PKBα* ^+/−^, and *PKBα* ^−/−^ isolated thymocytes using antibodies directed against either PKBα or phospho(Thr308)-PKB (PDK1 site). Actin was used as a loading control. B: Thymocytes were isolated from *PKBα* ^+/+^ and *PKBα* ^−/−^ thymic grafts and counted 4 weeks post grafting (left panel). Lymphocytes were isolated from thymus and spleen of lethally irradiated congenic recipient mice injected with either *PKBα* ^+/+^ or *PKBα* ^−/−^ fetal liver cells and counted 5 weeks post transplant (right panel). Error bars represent standard error of the mean; n≥5. C–D: Flow cytometric analysis of lymphocytes. C: Host-derived thymocytes developed in the *PKBα* ^+/+^ or *PKBα* ^−/−^ fetal thymi grafted under the kidney capsule of wild-type mice were isolated 4 weeks post-grafting and stained with cell surface markers for identification of early thymocyte subsets. D: Thymocytes developed from *PKBα* ^+/+^ or *PKBα* ^−/−^ fetal liver-derived HSC in lethally-irradiated wild-type congenic mice were isolated 5 weeks after reconstitution and stained with cell surface markers for identification of early thymocyte subsets. Representative density plots and histograms are shown. n≥5 (n = number of mice per genotype within the same experiment).

The thymus is composed of a heterogeneous population of cells, including thymocytes at various developmental stages and different stromal cells that are either hematopoietic, mesenchymal, or epithelial in origin. In thymocytes, PKBα was the main isoform located downstream of PDK1 since *PKBα* ^−/−^ thymocytes showed only minimally phosphorylated PKB levels at the PDK1 dependent-Thr308 residue (Figure 4A). *PKBα* expression was also observed in thymic epithelial cells (JG and GAH, unpublished), which are the most abundant component of the stromal compartment. Therefore, ablation of *PKBα* expression in either of these compartments could potentially account for the impairment in the transition from DN to DP thymocytes. To determine whether the observed phenotype was due to a lack of PKBα in non-hematopoietic stromal and/or in blood-borne cells, we next performed thymic grafting and fetal liver cell transfer experiments, respectively. In the first instance, we assessed the ability of *PKBα* ^−/−^ thymic stroma to support T cell development. For this purpose, embryonic day E15.5 thymi were isolated from both *PKBα* ^−/−^ and wild-type embryos. The fetal lobes were treated *in vitro* with deoxyguanosine for 6 days to deplete lymphoid cells, and then grafted under the kidney capsule of wild-type recipient mice. Four weeks post transplantation, the number of wild-type host-derived thymocytes developing within the *PKBα* ^−/−^ grafted thymic stroma was significantly reduced when compared to control tissue but regular thymocyte development was not affected (Figure 4B and 4C). In a second series of experiments, we evaluated the capacity of fetal liver derived-hematopoietic stem cells (HSC) from wild-type and *PKBα* ^−/−^ embryonic day E15.5 donors (CD45.2) to recapitulate normal thymopoiesis in wild-type thymic stromal environment of lethally-irradiated congenic (CD45.1) mice. Five weeks after reconstitution, the bone marrow chimeras had similar overall numbers of thymocytes and peripheral lymphocytes, irrespective whether they were derived from *PKBα* ^−/−^ or wild-type fetal liver cells (Figure 4B). Flow cytometric analyses further showed that *PKBα* ^−/−^ HSC were able to give rise to all thymocyte subsets (DN, DP, SP CD4^+^, and SP CD8^+^), but again both DN3 and ISP8 cells accumulated to the same extent as what had been observed in unmanipulated *PKBα* ^−/−^ mice (Figure 4D). Taken together, these data indicate that the accumulation of thymocytes during early T cell development observed in *PKBα*-deficient mice is the specific consequence of a lack of PKBα in lymphoid cells.

### The absence of PKBα in early thymocytes affects the expression of genes known to be regulated in thymocyte and T cell response processes, and in interferon signaling

**Figure 5:**
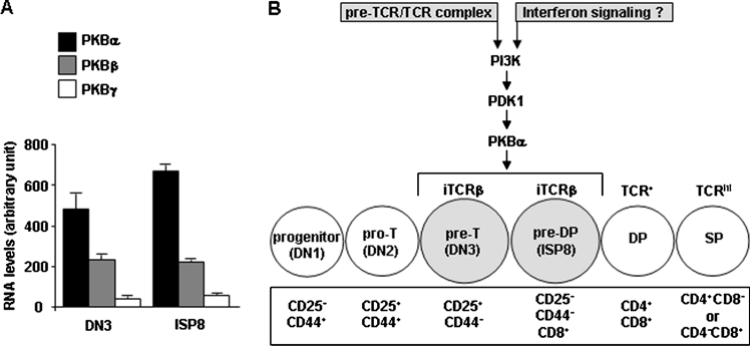
PKBα is the main isoform in DN3 and ISP8 thymocyte subsets. A: mRNA levels of PKBα, PKBβ, and PKBγ isoforms in DN3 and ISP8 thymocyte subsets. The expression data obtained following microarray analysis were corrected for GC-bias within oligos, allowing gene expression signals to be expressed on the same scale; this permits a semi-quantitative comparison of the expression of different genes. B: Proposed model of PKBα mediating PI3K signaling at the transition from DN to DP thymocyte subsets. iTCRβ and TCR refer to intracellular and surface expression of TCRβ, respectively.

As the developmental changes at early stages of thymocyte maturation appeared to be a cell-autonomous effect caused by the loss of *PKBα* expression, we next determined the gene expression profile in DN3 and ISP8 cells using Affymetrix microarrays. Expression data analysis of specific transcripts in wild-type DN3 and ISP8 sorted cells revealed that while PKBα was the main isoform in both of these thymocyte populations, PKBβ was expressed at a significantly lower level and PKBγ was present in an even lesser abundance (Figure 5A). These results suggest that PKBα is the main isoform expressed in DN3 and ISP8 thymocytes. Analyses of microarray data revealed that DN3 and ISP8 thymocytes were differently affected in their gene expression profiles by the absence of PKBα with only 5 genes being differentially expressed in both subpopulations (Tables 1 and 2). In the DN3 subset, the absence of PKBα resulted for example in a down-regulation of the chemokine (C-C motif) receptor 9 (*CCR9*), whose expression is known to be induced upon pre-TCR signaling [36]. This result suggests that the absence of PKBα potentially affects pre-TCR signaling in DN3. Moreover, the integrin alpha E epithelial-associated (*Itgae* or *CD103*) gene, that is known to be expressed in DN and whose product interacts with E-cadherin on thymic epithelial cells, was downregulated in the absence of PKBα. Furthermore, 8 genes whose expression was modified in *PKBα* ^−/−^ DN3 are typically induced by interferon and were systematically downregulated in cells lacking PKBα. These genes constituted 50% of all the genes whose expression was downregulated as a consequence of *PKBα* ablation in DN3 cells. In the ISP8 subset, several genes known to be induced in their expression upon TCR activation or involved in T cell activation were found to be downregulated in the absence of PKBα: the cell membrane glycoprotein *CD53* antigen, the lymphocyte antigen 6 complex locus A (*Ly6a*), the lymphocyte antigen 6 complex locus C (*Ly6c*), the T-cell specific GTPase (*TGTP*), or the MHC class II antigen (*H2-Aa*). In contrast, transcripts for other gene products known to act as negative regulators in TCR signaling, or in other pathways involved in T cell activation, were upregulated in the absence of PKBα, including the suppressor of cytokine signaling 3 (*SOCS3*), the cytotoxic T-lymphocyte-associated protein 4 (*CTLA-4*), or the immunoglobulin superfamily member *Igsf3*. Furthermore, some genes whose expression was upregulated in *PKBα* ^−/−^ ISP8, such as *PTEN*, *Notch3*, and one of its target genes *Dtx1*, have previously been shown to be involved in the transition from DN to DP thymocytes [37], [38]. Finally, 6 genes differentially expressed in *PKBα* ^−/−^ ISP8 are interferon-inducible in their expression and were systematically downregulated in cells lacking PKBα. These genes constituted 29% of all the genes whose expression was downregulated in *PKBα* ^−/−^ ISP8 cells.

**Table 1:** Genes with altered expression in PKBα^−/−^ FACS sorted DN3 thymocyte subset compared to PKBα^+/+^ cells.

	Gene name	Gene symbol	Affymetrix identification	GenBank accession number	Regulation (a)
pre-TCR signaling	chemokine (C-C motif) receptor 9	Ccr9	1427419_x_at	NM_009913	down regulated (×1.6)
	T cell receptor beta chain, clone library HK8.3-6F8	TCRb-V13	1444088_at	BE447255 (UniGene)	down regulated (×2.5)
T cell response/development process	integrin, alpha E, epithelial-associated	Itgae, CD103	1447541_s_at	NM_008399, NM_172944	down regulated (×1.6)
	phosphodiesterase 2A, cGMP-stimulated	Pde2a	1447707_s_at	NM_001008548	up regulated (×1.9)
Interferon-inducible	eukaryotic translation initiation factor 2-alpha kinase 2	Eif2ak2, PKR	1422006_at	NM_011163	down regulated (×1.7)
	interferon-induced protein with tetratricopeptide repeats 1	Ifit1, garg16	1450783_at	NM_008331	down regulated (×3.3)
	interferon inducible GTPase 1	Iigp1	1419042_at, 1419043_a_at	NM_021792	down regulated (×2.9)
	2′-5′ oligoadenylate synthetase 1A	Oas1a	1424775_at	NM_145211	down regulated (×2.2)
	2′-5′ oligoadenylate synthetase-like 2	Oasl2	1453196_a_at	NM_011854	down regulated (×2.0)
	radical S-adenosyl methionine domain containing 2 (*)	Rsad2, vig1, cig5, viperin	1436058_at	NM_021384	down regulated (×3.6)
	Receptor transporter protein 4 (*)	Rtp4, Ifrg28	1418580_at	NM_023386	down regulated (×1.6)
	ubiquitin specific peptidase 18	Usp18, UBP43, Ubp15	1418191_at	NM_011909	down regulated (×2.4)
Inflammation	scavenger receptor cysteine-rich type 1 protein CD163c-alpha precursor (*)	E430002D04Rik	1440808_x_at, 1455527_at	NM_172909	up regulated (×3.0)
	MAD homolog 7 (Drosophila)	Smad7	1423389_at	NM_001042660	up regulated (×1.6)
Signal transduction	G protein-coupled receptor, family C, group 5, member B	Gprc5b, Raig-2	1424613_at	NM_022420	up regulated (×2.3)
Transcription	RIKEN cDNA 1110051B16 gene	1110051B16Rik	1445710_x_at	NM_183389	down regulated (×2.1)
	predicted gene, EG622175	EG622175	1440202_at	XM_898168, XM_911074	up regulated (×1.7)
	myeloid leukemia factor 1 interacting protein	Mlf1ip	1428518_at	NM_027973	up regulated (×1.7)
	RAD54 homolog B (S. cerevisiae)	Rad54b	1434734_at	NM_001039556	up regulated (×1.7)
Other	cyclin-dependent kinase inhibitor 1A (P21)	Cdkn1a	1421679_a_at	NM_007669	down regulated (×1.7)
	plasma glutamate carboxypeptidase	pgcp, Hls2, Lal-1	1416441_at	NM_176073	down regulated (×1.6)
	aldo-keto reductase family 1, members C12 and C13	Akr1c12, Akr1c13	1422000_at	NM_013777	up regulated (×4.6)
	adaptor-related protein complex 3, mu 1 subunit	Ap3m1	1416374_at	NM_018829	up regulated (×1.7)
	HEAT repeat containing 1	Heatr1	1437965_at	NM_144835	up regulated (×1.8)
	ribonucleotide reductase M2	Rrm2	1448226_at	NM_009104	up regulated (×1.9)
	synaptotagmin-like 4	Sytl4	1417336_a_at	NM_013757	up regulated (×2.0)
	testis derived transcript	Tes	1424246_a_at	NM_011570, NM_207176	up regulated (×1.8)
	TCDD-inducible poly(ADP-ribose) polymerase	Tiparp	1452161_at	NM_178892	up regulated (×2.2)
	vitamin K epoxide reductase complex, subunit 1-like 1	Vkorc1l1	1429092_at	NM_001001327, NM_027121	up regulated (×1.7)
Unknown	PREDICTED: hypothetical protein LOC77994	2810055G20Rik	1445363_at, 1456787_at	BB451286 (UniGene)	down regulated (×2.4)
	cDNA sequence BC013672	BC013672	1439114_at, 1451777_at	NM_001081215	down regulated (×2.3)
	RIKEN cDNA 5830431A10 gene	5830431A10Rik	1436491_at	XR_002313	up regulated (×2.2)
	RIKEN cDNA A630023P12 gene (*)	A630023P12Rik	1455370_at	NM_173766	up regulated (×2.0)
	RIKEN cDNA B230342M21 gene	B230342M21Rik (LOC100637)	1444143_at	NM_133898	up regulated (×1.8)
	Mus musculus, clone IMAGE:3983821 (*)		1427820_at	BC021831	up regulated (×2.3)
	gb:BB337926		1440400_at	BB337926	up regulated (×1.7)

(a) P≤0.05, significant changes of ≥1.5-fold.

(*) genes modified in both DN3 and ISP8 subsets.

**Table 2:** Genes with altered expression in PKBα^−/−^ FACS sorted ISP8 thymocyte subset compared to PKBα^+/+^ cells.

	Gene name	Gene symbol	Affymetrix identification	GenBank accession number	Regulation (a)
TCR signaling	CD53 antigen	Cd53	1448617_at	NM_007651	down regulation (×1.8)
	suppressor of cytokine signaling 3	Socs3	1455899_x_at	NM_007707	up regulation (×2.0)
T cell response/development process	gap junction membrane channel protein alpha 1	Gja1, connexin43	1415800_at, 1437992_x_at	NM_010288	down regulation (×1.9)
	cytotoxic T-lymphocyte-associated protein 4	Ctla4, Cd152, Ly-56, Ctla-4	1419334_at	NM_009843	up regulation (×4.6)
	deltex 1 homolog (Drosophila)	Dtx1	1425822_a_at	NM_008052	up regulation (x 1.9)
	immunoglobulin superfamily, member 3	Igsf3, V7, Cd101, Igsf2	1455049_at	NM_207205	up regulation (×1.9)
	phospholipase C, beta 2, similar to phospholipase C, beta 2	LOC545451, Plcb2	1452481_at	NM_177568	up regulation (×1.9)
	Notch gene homolog 3 (Drosophila)	Notch3	1421964_at	NM_008716	up regulation (×1.9)
	phosphatidylinositol 3-kinase catalytic delta polypeptide	Pik3cd, p110δ	1453281_at	NM_001029837, NM_008840	up regulation (×1.6)
	Phosphatase and tensin homolog	Pten	1441593_at	NM_008960	up regulation (×2.2)
Interferon-inducible	Histocompatibility 2, class II antigen A, alpha	H2-Aa	1438858_x_at	NM_010378	down regulation (×1.9)
	lymphocyte antigen 6 complex, locus A	Ly6a, TAP, Sca1, Ly-6A.2, Ly-6A/E, Ly-6E.1	1417185_at	NM_010738	down regulation (×1.8)
	lymphocyte antigen 6 complex, locus C	Ly6c	1421571_a_at	NM_010741	down regulation (×2.2)
	radical S-adenosyl methionine domain containing 2 (*)	Rsad2, vig1, cig5, viperin	1436058_at	NM_021384	down regulation (×2.9)
	Receptor transporter protein 4 (*)	Rtp4, Ifrg28	1418580_at	NM_023386	down regulation (×2.3)
	T-cell specific GTPase	Tgtp, Gtp2; Mg21	1449009_at	NM_011579	down regulation (×1.8)
Inflammation	scavenger receptor cysteine-rich type 1 protein CD163c-alpha precursor (*)	E430002D04Rik	1455527_at	NM_172909	up regulation (×2.2)
Signal transduction	guanine nucleotide binding protein (G protein), gamma 12	Gng12	1421947_at	NM_025278	down regulation (×1.6)
	tubulin, beta 3	Tubb3	1415978_at	NM_023279	down regulation (×1.8)
	BMP and activin membrane-bound inhibitor, pseudogene (Xenopus laevis)	Bambi-ps1	1456178_at	BF730112 (UniGene)	up regulation (×3.4)
Transcription	nuclear factor I/X	Nfix	1436364_x_at	NM_010906	down regulation (×1.8)
	gb:BG073921	AF4/FMR2 family, member 1, Aff1, Af4, Rob, Mllt2h	1444937_at	NM_133919	up regulation (×2.3)
Other	Acyl-CoA thioesterase 6	Acot6	1428803_at	NM_172580	down regulation (×1.6)
	expressed sequence C85492	C85492, Ago61	1436489_x_at	NM_153540	down regulation (×2.6)
	cyclin D2	Ccnd2	1430127_a_at, 1434745_at	NM_009829	down regulation (×2.0)
	cytoplasmic FMR1 interacting protein 1	Cyfip1	1416329_at	NM_011370	down regulation (×2.2)
	similar to Zinc finger DHHC domain containing protein 6 (H4 homolog)	LOC433204, Zdhhc6	1441611_at	NM_001033573, NM_025883	down regulation (×1.8)
	region containing histocompatibility 2, Q region locus 9 and locus 7	LOC630509, LOC674192	1418536_at		down regulation (×1.9)
	protein disulfide isomerase associated 5	Pdia5	1424650_at	NM_028295	down regulation (×1.6)
	solute carrier family 22 (organic cation transporter), member 3	Slc22a3, EMT; EMTH; OCT3	1420444_at	NM_011395	down regulation (×2.0)
	testis derived transcript	Tes, TESS, Tes1, Tes2, testin2, D6Ertd352e	1460378_a_at	NM_011570, NM_207176	down regulation (×1.7)
	RIKEN cDNA 2310032D16 gene	2310032D16Rik, Prei4	1458701_at	NM_001042671, NM_001042672, NM_028802	up regulation (×2.2)
	arginine-tRNA-protein transferase 1	Ate1	1420652_at	NM_001029895, NM_013799	up regulation (×1.9)
	RIKEN cDNA 5730405I09 gene, cyclin Y	Ccny	1441283_at	NM_026484	up regulation (×3.6)
	CDC14 cell division cycle 14 homolog B (S. cerevisiae)	Cdc14b	1437070_at	NM_172587	up regulation (×1.7)
	cytidine monophospho-N-acetylneuraminic acid hydroxylase	Cmah	1421214_at, 1447019_at	NM_007717	up regulation (×2.1)
	fibulin 1	Fbln1	1451119_a_at	NM_010180	up regulation (×2.1)
	hepatoma-derived growth factor, related protein 3	Hdgfrp3, HRP-3	1423252_at	NM_013886	up regulation (×1.7)
	similar to solute carrier family 28 (sodium-coupled nucleoside transporter), member 2	LOC381417, Slc28a2, cnt2	1450639_at	NM_172980	up regulation (×2.2)
	nudix (nucleoside diphosphate linked moiety X)-type motif 21	Nudt21	1455966_s_at	NM_026623	up regulation (×2.0)
	palmitoyl-protein thioesterase 1	Ppt1, PPT, CLN1, INCL	1420015_s_at	NM_008917	up regulation (×1.8)
	Sfi1 homolog, spindle assembly associated (yeast)	Sfi1	1426787_at	NM_030207	up regulation (×2.1)
	gb:BB201882	zinc finger RNA binding protein (zfr)	1443353_at	NM_011767	up regulation (×2.7)
Unknown	RIKEN cDNA 2610019E17 gene	2610019E17Rik	1419798_at	AK011460	down regulation (×1.5)
	RIKEN cDNA 1700054N08 gene	1700054N08Rik	1424796_at	NM_028536	up regulation (×1.8)
	RIKEN cDNA 5830416P10 gene	5830416P10Rik	1453244_at	AK017935	up regulation (×2.5)
	RIKEN cDNA A130022J15 gene	A130022J15Rik	1433671_at	NM_175313	up regulation (×1.6)
	RIKEN cDNA A630023P12 gene (*)	A630023P12Rik	1455370_at	NM_173766	up regulation (×2.7)
	CDNA sequence BC031575, mRNA, RIKEN cDNA 4921513D23 gene	BC031575, 4921513D23Rik	1447110_at	NM_153549	up regulation (×2.3)
	gb:BG086638		1420312_s_at	BG086638	up regulation (×4.0)
	Mus musculus, clone IMAGE:3983821 (*)		1427820_at	BC021831	up regulation (×2.1)
	gb:AW546720		1446882_at	AW546720	up regulation (×1.7)

(a) P≤0.05, significant changes of ≥1.5-fold.

(*) genes modified in both DN3 and ISP8 subsets.

## Discussion

### The deletion of *PKBα* leads to a reduced size of the thymus in mouse neonates, which is attributed to hypocellularity

The regulation of both cell number and volume contributes to the establishment of organ size. A number of studies have implicated the PI3K signaling pathway, and more specifically PKB, in determination of cell, organ, and body size. Tissue-specific activation of this pathway, either by expressing active PI3K or PKB or by deleting PTEN, results in an increased organ weight, a finding often associated with enlarged cell volume [39]–[41]. In contrast, the ablation of a single PKB isoform causes a reduction in the size of the animal and/or specific organs. For instance, deletion of *PKBα* leads to a 30% reduction of body weight [26], [28], [34], while ablation of *PKBγ* specifically causes a significant reduction in brain tissue due to reduced cell number and size [29], [32]. In this study, we report a disproportionally reduced thymic size in *PKBα* ^−/−^ neonates that consistently show reduced thymic cellularity, the extent of which was somewhat variable. This decrease was not due to an increase in thymocyte apoptosis. Contrary to this latter result, a previous study reported an increase in spontaneous apoptosis among *PKBα* ^−/−^ thymic cells of adult mice [26] yet, this observation was not linked to any reduced organ size. This apparent discrepancy between the two studies may possibly arise from a variation in the age of the mice analyzed and/or from differences in the genetic background; while the genetic background of the *PKBα* ^−/−^ mice in our study was statistically above 90% C57Bl/6, in the study reported by Chen *et al.* it was an equal mix of C57Bl/6 and 129 R1.

The lymphoid component of the thymus is not self-renewing and must be continually reseeded by fetal liver or adult bone marrow derived thymic progenitor cells. As such, the decrease in thymocyte numbers observed in *PKBα* ^−/−^ neonates could be lymphoid cell autonomous and relate to a reduction in either the absolute number or the efficiency of thymic progenitor cells. Alternatively, or additionally, the thymic cellularity could be affected by a defective thymic microenvironment in *PKBα* ^−/−^ neonates. Indeed, *PKBα*-deficient thymic grafts displayed a decrease in thymocyte number, which was not associated with impaired T cell development. In addition, in some of the *PKBα* ^−/−^ neonates, thymic sections analyzed using hematoxylin and eosin staining as well as immunohistology displayed disorganized cortical/medullary epithelial cell compartment (Figure S3). However, neither cellularity nor morphology was abnormal in thymi of adult *PKBα* ^−/−^ mice nor in *PKBβ* ^−/−^ and *PKBγ* ^−/−^ neonatal thymi. We speculate that the hypocellularity observed in *PKBα* ^−/−^ neonatal thymi could be due to a delay in thymic development, possibly and partly originating from a defective microenvironment within the thymus at early stages.

### The lack of PKBα in lymphoid cells leads to an accumulation of thymocyte subsets at the DN to DP transition in early T cell development

Alteration in specific components of the PI3K signaling pathway, such as PDK1, leads to an impaired transition from DN to DP thymocytes, suggesting an essential role of factors downstream of PDK1 in T cell development. PKB is the most important mediator of the PI3K signaling and, from our data, PKBα is the main functional PKB isoform positioned downstream of PDK1 in thymocytes. Our study highlights an accumulation of *PKBα* ^−/−^ DN3 and ISP8 thymocyte subsets. We attribute this accumulation to a cell-autonomous lack of PKBα within the T lymphoid component of the thymus and concurrently exclude a contribution by *PKBα*-deficient thymic stroma to this finding. While the deletion of *PKBα* does not prevent further maturation to the SP stages, our results indicate that PKBα is important in the transition from DN to DP. This effect is not due to impaired TCRβ chain expression, even though we observed downregulated expression of one of the numerous TCRβ-V segments (*Vβ13*) in *PKBα* ^−/−^ DN3 thymocytes. Furthermore, the surface expression of the α chain of the interleukin-2 receptor (CD25) was increased in the *PKBα* ^−/−^ DN3 subset. While with our current knowledge, we cannot relate this observation to the phenotype observed, this increased CD25 surface expression has also been reported in DN3 cells lacking PDK1 [17]. Our data suggest that the α isoform of PKB is an important effector of PDK1 in the transition from DN to DP subsets, which constitutes a critical step during T cell development. Interestingly, in view of the reduced percentage of CD25^−^CD44^+^c-kit^−^ thymocytes in *PKBα* ^−/−^ thymi, PKBα could also affect a subpopulation of cells within the thymus that is positive for CD44 surface expression but not (yet) committed to the T cell lineage.

While one could hypothesize that the distinct phenotypes reported in *PKBα*, *PKBβ*, and *PKBγ* mutant mice are due to specific and distinct functions of the PKB isoforms, it could be equally well argued that these differences are merely due to a loss of an abundant isoform, which leads in a specific tissue to a reduction of total PKB below a critical level. Based on our data concerning differential expression levels of PKBα, PKBβ, and PKBγ in early thymocyte subsets, we predict that a combined deletion of *PKBα* and *PKBβ* would lead to a more extensive block during early T cell development compromising thymocyte maturation further. Mice lacking both *PKBα* and *PKBβ*, however, die at birth with multiple defects [31]. Moreover, while complete deletion of *PDK1* in early thymocytes arrests their progression to mature T cells, reduced PDK1 expression to 10% of normal levels still allows T cell development [17]. Therefore, the residual PKB activity present in *PKBα* ^−/−^ thymocytes might be sufficient to permit thymocytes to progress to mature T cells despite accumulation of early thymocyte subsets at the DN to DP transition. Alternatively and in view of the potential role attributed to the serine/threonine kinase S6K downstream of PDK1 [42], we suspect that PKB and S6K could compensate for each other during thymocyte development. This contention is further supported by the finding that single S6K mutant mice fail to reveal a defect in T cell development [43], [44].

The signal transduction pathways that control thymocytes are often recapitulated in mature T cells. From our data, a number of genes whose expression is modulated upon the loss of *PKBα* are known to be involved in pre-TCR and/or TCR signaling and T cell activation. The presented results hence suggest that the deletion of *PKBα* affects the pre-TCR signaling in early thymocytes. Interestingly, several recent reports show a significant role of the PI3K pathway in the pre-TCR controlled developmental transition of DN to DP thymocytes. For instance, TCRβ-deficient mice activated by anti-CD3ε to mimic pre-TCR signals reveal a significant impairment of their DN to DP progression in the absence of p85α (the major regulatory subunit of PI3K) [45]. Moreover, only immature thymocytes with a functional pre-TCR display evidence for PDK1 activation *in situ* [42]. Finally, deletion of *PTEN* in T cells or expression of a constitutively active mutant of PKB can substitute for the pre-TCR signals required for thymocyte maturation [38], [46]. PTEN expression is upregulated in ISP8 thymocytes lacking PKBα. Besides pre-TCR, the Notch pathway controls T cell development during the progression from DN to DP subsets. More particularly, Notch3 is normally expressed in DN thymocytes and downregulated across the DN to DP transition [47]. Mice expressing the intracellular domain of Notch3 in thymocytes are characterized by the accumulation of DN3 cells and the increased expression of CD25 [48]. Strikingly, in *PKBα* ^−/− ^ISP8 thymocytes, we observed upregulation of *Notch3* expression together with *Dtx1*, one of its target genes. Nonetheless, it remains to be investigated whether the upregulated *Notch3* expression in *PKBα* ^−/− ^ISP8 cells is functionally linked to the accumulation of DN3 and ISP8 thymocytes and the increased CD25 surface expression among DN3 thymocytes.

A number of genes whose expression is known to be inducible by interferon were systematically downregulated in *PKBα* ^−/−^ DN3 and/or ISP8 cells. Interestingly, the PI3K signaling pathway was shown to be activated by both interferon-α and interferon-γ and to control important regulatory transcriptional events [49]. For instance, PI3K-PKB pathway plays an important role in the phosphorylation of STAT1 (the main transcriptional effector of interferon-γ) and in subsequent activation of gene expression in response to interferon-γ [50]. In addition, PI3K is able to mediate responses to interferon by acting independently of STAT and represents an alternative pathway to the well studied Jak-STAT pathway [49]. Moreover, both interferon-α and interferon-γ induce a rapid phosphorylation of S6K, which subsequently phosphorylates the S6 ribosomal protein [49]; this activation was shown to be dependent on PI3K and the mammalian target of rapamycin (mTOR). PKB is involved in the activation of S6K via an indirect activation of mTOR. Significantly, *PDK1*-deficient early thymocytes lack phosphorylated S6 [17]. The functional roles of the PI3K pathway in mediating interferon signals in various cell types, especially thymocytes, remain undefined. Our results indicate that molecules typically induced as a consequence of interferon signaling are involved in the DN to DP transition during T cell development in a PKBα-dependent manner.

During the preparation and the revision process of our manuscript, two publications have reported that the combination of a T cell-specific *PKBα* deletion with a complete or a T cell-specific *PKBβ* deletion leads to a more extensive block at the DN to DP transition [51], [52]. The additional ablation of *PKBγ* further compromises T cell maturation beyond the DN stages [51]. Moreover, one of these reports shows that PKB*α* is the most highly expressed isoform in the DN1-4 and DP subsets [52], which is in line with and expands our expression analysis for DN3 and ISP8 thymocytes. Interestingly, while the absence of PKBα alone did not result in apparent changes in proliferation and apoptosis (our study), ablation of both *PKBα* and *PKBβ* (i) interferes with the differentiation of DN3 [51], [52], which was attributed to apoptosis partially due to decreased cellular growth and metabolism [52], (ii) inhibits the proliferation of DN4 cells [51], and (iii) reduces the survival of DP thymocytes [51]. Furthermore, combined ablation of all three PKB isoforms inhibits the survival of all the DN thymocytes [51]. Finally, these two publications could show that pre-TCR signals activate PKB [51], [52], which supports one of the conclusions from our microarray analysis. Together with our study, these results further highlight the crucial role of PKB during early T cell development and the fact that PKBβ and, to a lesser extent, PKBγ isoforms compensate for PKBα in this process.

### Conclusion

In conclusion our data show that PKBα, one of the three PKB isoforms, plays a crucial role in thymic development and represents a key effector of the PI3K signaling pathway in early thymocyte development. Our results further indicate that PKBα not only mediates signals downstream of the pre-TCR but also regulates the expression of genes typically controlled by interferon signaling during a critical transition in T cell development. We suggest that PKBα could account, at least in part, for the block in early T cell development reported in mice deficient for components of the PI3K pathway upstream of PKB. Our results are summarized in Figure 5B. The critical question now is to identify the PKB targets that function at this checkpoint in a phosphorylation-dependent fashion.

## Materials and Methods

### Mice

Mice were group-housed with 12 hour-dark/light cycles and free access to food and water, in accordance to the Swiss Animal Protection Ordinance. All procedures were conducted with approval of the appropriate authorities. *PKBα* ^−/−^, *PKBβ* ^−/−^, and *PKBγ* ^−/−^ mice were generated in our laboratory and previously described [32], [34], [53]. B6 Ly5.1 and Fox8 rosa26 mouse lines were obtained from The Jackson Laboratory (Bar Harbor, ME, USA).

### Western-Blot analysis

Tissues were homogenized in lysis buffer (50 mM Tris-HCl pH 7.5, 150 mM NaCl, 1% Nonidet P-40, 1 mM benzamidine, 1 mM phenylmethylsulfonyl fluoride, and 2 µM microcystin-LR (Alexis Corporation, San Diego, CA, USA), 1 mM sodium pyrophosphate, 10 mM NaF and 0.1 mM sodium orthopervanadate) and debris removed by two centrifugation steps at 16 000 g for 10 minutes at 4°C. Protein concentration was determined using the Bradford assay (Bio-Rad Laboratories, Hercules, CA, USA) with BSA as standard. Fifty µg of protein extracts were separated by 10% SDS-PAGE and transferred onto PVDF membrane (Millipore, Billerica, MA, USA) by electroblotting. Membranes were blocked with 5% BSA in TBST (50 mM Tris-HCl pH 7.5, 150 mM NaCl, and 0.1% Tween 20), incubated for 16 hours at 4°C with the primary antibody and 1 hour at room temperature with horseradish peroxidase-conjugated anti-rabbit or anti-mouse secondary antibodies, and analyzed using enhanced chemiluminescence reagents (Amersham Biosciences, Piscataway, NJ, USA). PKB isoform-specific antibodies obtained by immunizing rabbits with isoform-specific peptides have already been reported [34]. Antibodies against phospho Thr308-PKB (the PDK1 site) and pan-actin were purchased from Cell Signalling Technologies (Danvers, MA, USA) and NeoMarkers (Fremont, CA, USA), respectively.

### TUNEL assay

Mouse thymi were fixed in formalin (10% v/v) for 16 hours at 4°C. After dehydration in ethanol, samples were embedded in paraffin, cut into 5 µm-thick sections, and treated with 20 µg/ml proteinase K for 10 minutes at 37°C. Endogenous peroxidase was inactivated with 3% H_2_O_2_ in methanol for 30 minutes at room temperature. The sections were incubated in terminal deoxynucleotidyl transferase (TdT) buffer for 15 minutes at room temperature and TdT and biotinylated dUTP for 1 hour at 37°C. Washing with 1X SSC (0.15 M NaCl, 0.015 M sodium citrate) was used to stop the reaction. The Vectastain ABC kit (Vector Laboratories, Burlingame, CA, USA) was used for color development as described by the manufacturer. For quantification, 5 fields in each of 3 sections were counted for TUNEL-positive cells.

### Flow cytometric analysis and FACS sorting

Two million lymphocytes in suspension were stained at 4°C for 20 minutes in FACS buffer (PBS and 2% FCS) with fluorescein isothiocyanate (FITC)-, phycoerythrin (PE)-, Cy5-, and/or biotin-conjugated antibodies to cell surface molecules. Biotinylated antibodies were visualized with streptavidin-Cy5. For labeling of thymocyte precursors, cells were stained with FITC-CD25, PE-CD44, and biotin-CD4, CD8, TCRβ, TCRγδ, CD19, B220, CD11b, CD11c, Gr-1, and NK1.1. Cy5-negative precursor cells, corresponding to lineage-negative cells, were analyzed for expression of CD25 and CD44. Cells were stained with FITC-CD3, PE-CD4, and Cy5-CD8 to label later stages. For labeling of peripheral lymphocytes, cells were isolated from the spleen, depleted of red blood cells, and stained with PECy7-CD19 and Cy5-CD3. For intracellular staining, lymphocytes labeled with cell surface markers were incubated for 16 hours at 4°C in fixation buffer (BD Biosciences, San Jose, CA, USA) and processed in permeabilization buffer (BD Biosciences). For the analysis of thymocyte apoptosis, 10^6^ cells were stained at 4°C for 20 minutes in annexin binding buffer (Vybrant apoptosis assay kit #3, Molecular Probes, Eugene, OR, USA) with FITC-annexin V and propidium iodide (PI) according to the manufacturer's instructions. For flow cytometric analysis, labeled thymocytes were washed with FACS buffer, permeabilization buffer (when intracellular staining), or annexin binding buffer (when annexin V-PI staining) and analyzed on a FACSCalibur (Becton Dickinson, Franklin Lakes, NJ, USA). Data were processed with Cell Quest Pro (BD Biosciences). For FACS sorting, labeled thymocytes were washed with FACS buffer, filtered on a 40 µm-nylon membrane, and sorted on the flow sorter MoFlo (DakoCytomation, Baar, Switzerland).

### Bone marrow transplant and thymic grafting experiments

For bone marrow transplant experiments, fetal liver from *PKBα* ^+/+^ and *PKBα* ^−/−^ E15.5 embryos (CD45.2) were dissected and disrupted to single cell suspension by passages through a G25-syringe. The resultant suspension was layered over Ficoll and spun down for 25 minutes at 2 000 g. After removing the buffy coat, the fetal liver cells were washed, counted, and resuspended at 5×10^6^ cells/ml. Bone marrow chimeras were generated by intravenous injection of 10^6^ fetal liver cells into lethally irradiated (2×550 Rad) 4 week-old congenic recipient mice (CD45.1) on a C57Bl/6 background (B6 Ly5.1). The donor derived-lymphocyte populations were analyzed by flow cytometry 5 weeks post transplant. For grafting experiments, fetal thymic lobes from *PKBα* ^+/+^ and *PKBα* ^−/−^ E15.5 embryos were dissected and depleted of thymocytes by 6 day-treatment with 1.35 mM deoxyguanosine. Donor thymic stroma were then subrenally engrafted into 4 week-old Fox8 rosa26 recipient mice. The grafts were analyzed by flow cytometry 4 weeks post grafting.

### RNA extraction and microarray experiment

DN3 and ISP8 thymocyte subsets were sorted by FACS from 4 *PKBα* ^−/−^/wild-type littermate pairs. The same number of DN3 or ISP8 cells was sorted (7 000 to 25 000 cells) within a *PKBα* ^−/−^/wild-type pair. Total RNA was extracted using PicoPure™ RNA isolation kit (Arcturus, Sunnyvale, CA, USA) according to manufacturer's instructions. RNA quality was controlled using the 2100 Bioanalyser (Agilent Technologies, Santa Clara, CA, USA). Total RNA was amplified and labeled using the Affymetrix 2-cycle 3′ labeling kit according to manufacturer's instructions. After fragmentation, 10 µg cRNA was hybridised to mouse genome 430 2.0 GeneChips (Affymetrix, Santa Clara, CA, USA). After scanning the Genechips in an Affymetrix 2500 scanner, transcript expression values were estimated using the GC-RMA function provided by Refiner 3.1 (Genedata, Basel, Switzerland) and statistical analysis was performed using Analyst 3.1 (Genedata). Genedata's implementation of GC-RMA includes the generation of an Affymetrix detection P-value. A gene was considered to be reliably detected if it had a detection P-value≤0.04 (Affymetrix default, marginal calls ignored) in at least 2/3 of the biological replicates of a condition. A power analysis of our experimental design showed we could expect to have a power of 0.8 to distinguish samples differing by 1.5-fold with a normalised standard deviation less than 0.461 and it could resolve differences of 2-fold (power of 0.8) when the normalised standard deviation was less than 0.613. We selected genes that were significantly (paired t-test P≤0.05) modified by ≥1.5-fold between *PKBα* ^−/−^ and the corresponding control in at least three of the four pairs. Only genes with expression data above 20 in at least one of the conditions within a pair and in at least 3 pairs are displayed. The microarray data have been deposited in the Gene Expression Omnibus of NCBI (accession number: GSE7875).

### Statistical analysis

Data are provided as arithmetic mean±standard error of the mean and tested for significance using one-way analysis of variance (ANOVA). Only results with a P value of ≤0.05 (*) were considered statistically significant.

### Note

Materials and Methods related to Figures in Supporting Information can be found in “Materials and Methods S1”.

## Supporting Information

Figure S1 The deletion of *PKBα* does not affect T cell number in adult mice. A: The weight of freshly dissected thymi was measured in *PKBα* ^+/+^ and *PKBα* ^−/−^ adult mice and expressed as ratio to body weight. B: Thymocytes were isolated from *PKBα* ^+/+^ and *PKBα* ^−/−^ adult mice and counted; cell number was expressed as ratio to body weight. n≥3 (n = number of mice analyzed per genotype). Error bars represent standard error of the mean.
(0.87 MB TIF)

Figure S2 FACS analysis of different thymocyte subsets. A: Density plots show the main thymocyte subsets from *PKBα* ^+/+^ and *PKBα* ^−/−^ mice: DN (CD4^−^CD8^−^), DP (CD4^+^CD8^+^), SP CD4^+^ (CD4^+^CD8^−^), and SP CD8^+^ (CD4^−^CD8^+^). The results shown are representative of three independent experiments on 4 to 6 week-old mice. B: Histograms show the intracellular protein expression of TCRβ (iTCRβ) in DN4 thymocytes from *PKBα* ^+/+^ and *PKBα* ^−/−^ mice. C–D: Histograms show BrdU incorporation (C) or annexin V staining (D) in specific thymocyte subsets from *PKBα* ^+/+^ and *PKBα* ^−/−^ mice.
(5.85 MB TIF)

Figure S3 The deletion of *PKBα* tends to lead to disorganized thymic structures in neonates. A–B: Hematoxylin and eosin staining of 5 µm-thick sections from formalin-fixed paraffin-embedded mouse thymi from (A) *PKBα* ^+/+^ and *PKBα* ^−/−^ littermates at neonatal and adult ages and (B) *PKBβ* ^+/+^, *PKBβ* ^−/−^, *PKBγ* ^+/+^, and *PKBγ* ^−/−^ neonatal littermates. The bar shown on the pictures represents 200 µm. C: Immunohistochemical staining of mouse thymi from *PKBα* ^+/+^ and *PKBα* ^−/−^ littermates at neonatal age using anti-cytokeratin-8 and anti-cytokeratin-5 antibodies. (*) keratin free regions, (m) medullary regions, (c) cortical regions, (arrow) globular medullary epithelial cells. Images acquired using a 40x objective lens, image field is originally 230 µm.
(9.96 MB TIF)

Materials and Methods S1 (0.02 MB DOC)
