# Supplementary figures and images for: Deletion of PKBα/Akt1 Affects Thymic Development
Source: PLoS One. 2007 Oct 3;2(10):e992. doi: 10.1371/journal.pone.0000992 (PMC1991598; doi:10.1371/journal.pone.0000992)

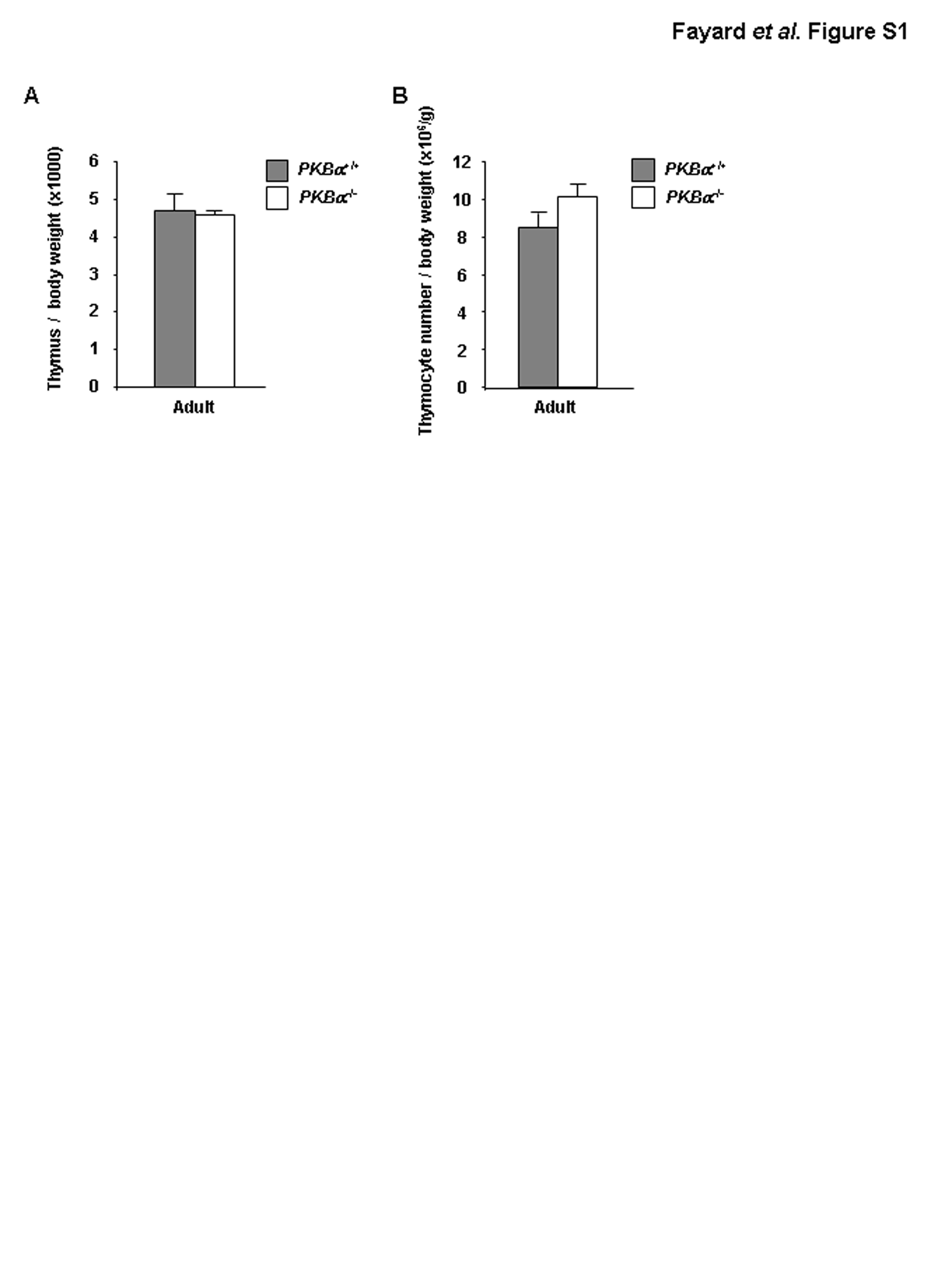

Supplement: Figure S1 — The deletion of PKBα does not affect T cell number in adult mice. A: The weight of freshly dissected thymi was measured in PKBα +/+ and PKBα −/− adult mice and expressed as ratio to body weight. B: Thymocytes were isolated from PKBα +/+ and PKBα −/− adult mice and counted; cell number was expressed as ratio to body weight. n≥3 (n = number of mice analyzed per genotype). Error bars represent standard error of the mean. (0.87 MB TIF) [file pone.0000992.s001.tif]

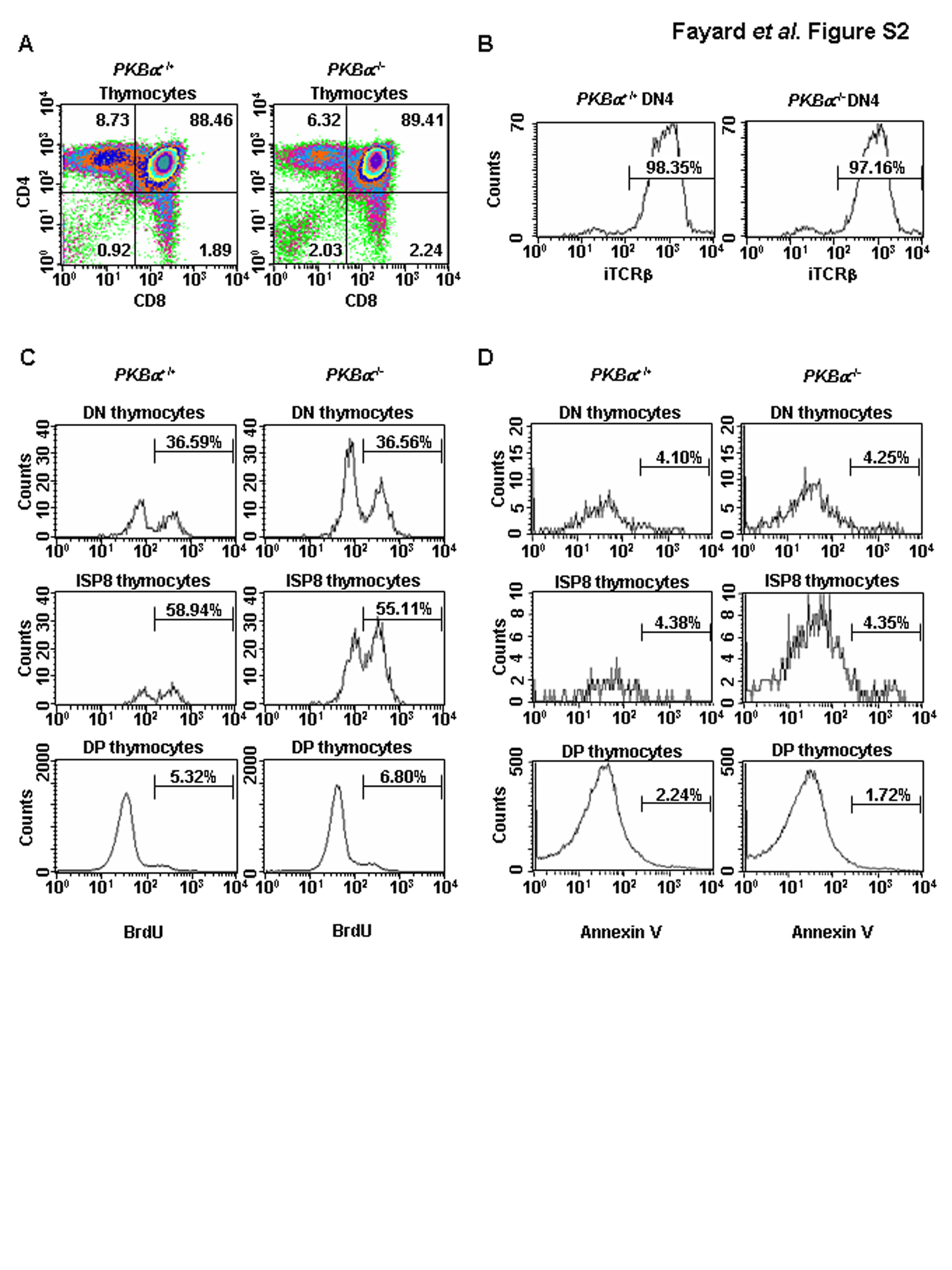

Supplement: Figure S2 — FACS analysis of different thymocyte subsets. A: Density plots show the main thymocyte subsets from PKBα +/+ and PKBα −/− mice: DN (CD4−CD8−), DP (CD4+CD8+), SP CD4+ (CD4+CD8−), and SP CD8+ (CD4−CD8+). The results shown are representative of three independent experiments on 4 to 6 week-old mice. B: Histograms show the intracellular protein expression of TCRβ (iTCRβ) in DN4 thymocytes from PKBα +/+ and PKBα −/− mice. C–D: Histograms show BrdU incorporation (C) or annexin V staining (D) in specific thymocyte subsets from PKBα +/+ and PKBα −/− mice. (5.85 MB TIF) [file pone.0000992.s002.tif]

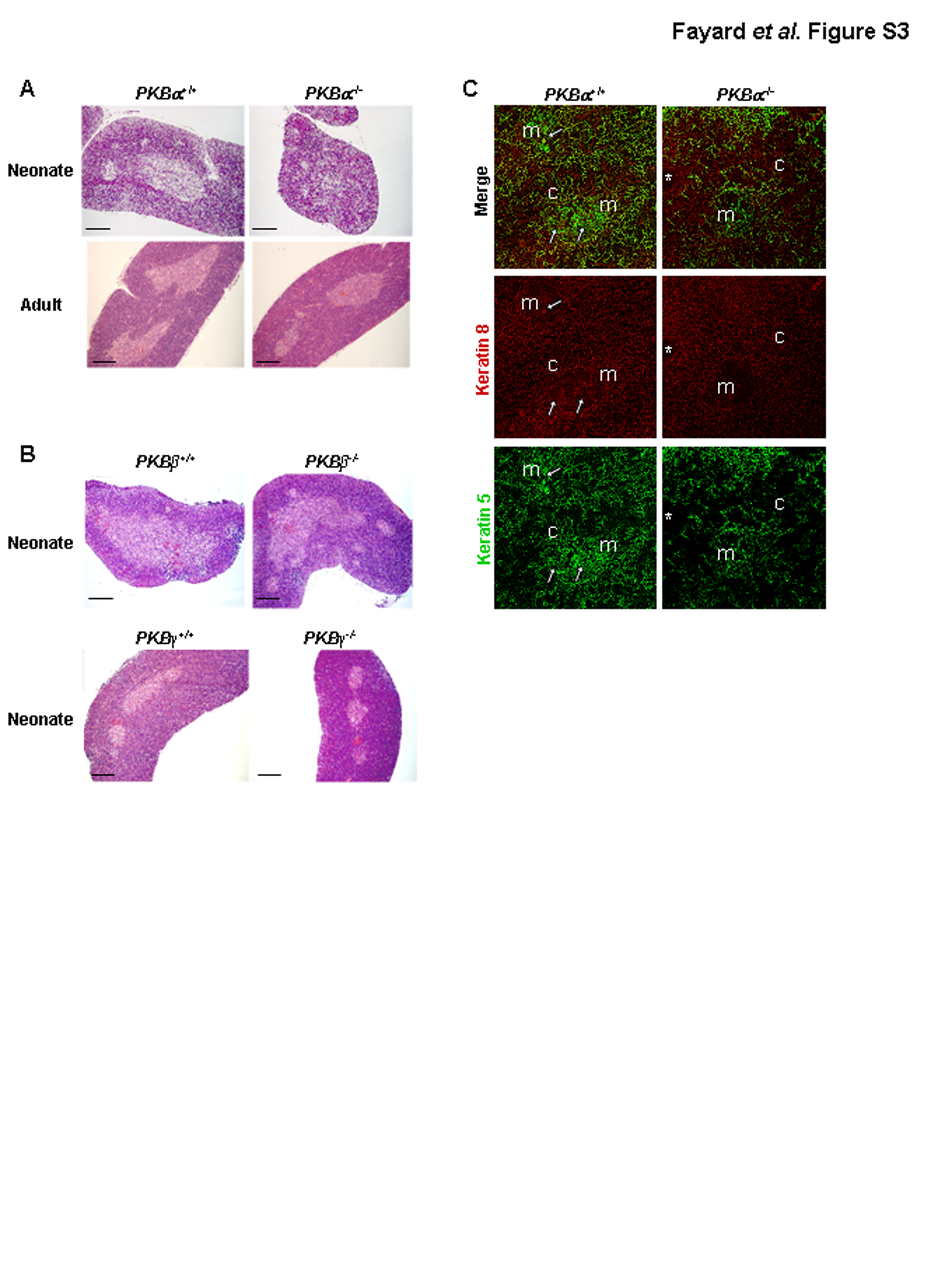

Supplement: Figure S3 — The deletion of PKBα tends to lead to disorganized thymic structures in neonates. A–B: Hematoxylin and eosin staining of 5 µm-thick sections from formalin-fixed paraffin-embedded mouse thymi from (A) PKBα +/+ and PKBα −/− littermates at neonatal and adult ages and (B) PKBβ +/+, PKBβ −/−, PKBγ +/+, and PKBγ −/− neonatal littermates. The bar shown on the pictures represents 200 µm. C: Immunohistochemical staining of mouse thymi from PKBα +/+ and PKBα −/− littermates at neonatal age using anti-cytokeratin-8 and anti-cytokeratin-5 antibodies. (*) keratin free regions, (m) medullary regions, (c) cortical regions, (arrow) globular medullary epithelial cells. Images acquired using a 40x objective lens, image field is originally 230 µm. (9.96 MB TIF) [file pone.0000992.s003.tif]
