# Supplementary material for: Deletion of PKBα/Akt1 Affects Thymic Development
Source: PLoS One. 2007 Oct 3;2(10):e992. doi: 10.1371/journal.pone.0000992 (PMC1991598; doi:10.1371/journal.pone.0000992)
Supplement: Materials and Methods S1 — (0.02 MB DOC) [file pone.0000992.s004.doc]

**Materials and Methods S1**

**BrdU labeling**

Mice were injected intraperitoneally with 5’-bromo-2’-deoxyuridine (BrdU, Sigma, Buchs, Switzerland) (1 mg/100 µl) and sacrificed 4 hours later. Two million thymocytes in suspension were stained at 4°C for 20 minutes in FACS buffer (PBS and 2% FCS) with PE-CD4, PECy7-CD3, and Cy5-CD8. Cells were then fixed, permeabilized, and stained with FITC-labeled anti-BrdU antibody (Becton Dickinson). Labeled thymocytes were washed with permeabilization buffer and analyzed by flow cytometry.

**Annexin V labeling**

Two million thymocytes in suspension were stained at 4°C for 20 minutes in FACS buffer (PBS and 2% FCS) with PE-CD4, PECy7-CD3, and Cy5-CD8. Cells were then stained with FITC-annexin V (Molecular Probes) according to the manufacturer’s instructions and analyzed by flow cytometry.

**Immunohistology**

Mouse thymi were embedded in OCT compound, snap frozen and cut into 6 µm-thick cryosections. Sections were blocked at room temperature for 10 minutes with PBS supplemented with 10% FCS. Primary monoclonal antibodies used were anti-cytokeratin-8 (TROMA-1, developmental studies hybridoma database) and anti-cytokeratin-5 (Covance, Princeton, NJ, USA). Secondary antibodies used for detection were goat anti-rat Alexa 555 and goat anti-rabbit Alexa 488 (Molecular Probes). Isotype control antibodies were used for all experiments. Images were captured on a Zeiss LSM 510 Meta Laser Scanning Confocal Microscope system (Carl-Zeiss, Feldbach, Switzerland). Image overlays were colored by computer-assisted management of confocal microscopy data generated with Zeiss LSM 510 software version 3.2.
